# Recommendations to facilitate managers’ compliance with quality standards at primary health care clinics

**DOI:** 10.4102/curationis.v42i1.1984

**Published:** 2019-04-23

**Authors:** Lebuile J. Mogakwe, Hafisa Ally, Nomasonto B.D. Magobe

**Affiliations:** 1 Department of Nursing Science, University of Johannesburg, Johannesburg, South Africa

**Keywords:** facilitating, managers, compliance, quality standards, primary health care clinics

## Abstract

**Background:**

The Republic of South Africa (RSA) is shifting towards universal health coverage and a unified health system. This milestone can be achieved through the implementation of National Health Insurance (NHI). To employ NHI, health establishments in the country are compelled to comply with quality standards. The non-compliance with quality standards at primary health care (PHC) clinics within a district in Gauteng, which was verified by quality standards’ audit reports, prompted an intervention. No prior research aimed at facilitating managers’ compliance with quality standards has been conducted within the context under study. This research gap necessitated an exploration on how managers’ compliance to quality standards at PHC clinics within a district in Gauteng could best be facilitated.

**Objectives:**

To describe recommendations to facilitate managers’ compliance with quality standards at PHC clinics within a district in Gauteng.

**Method:**

A qualitative, exploratory, descriptive and contextual research design was used in this study. Semi-structured, individual interviews were conducted.

**Results:**

The recommendations to facilitate managers’ compliance with quality standards at PHC clinics within a district in Gauteng were described. However, for the purpose of this article, only the recommendations seeking to address challenges with management practices as a reason for non-compliance with quality standards at PHC clinics will be discussed. These recommendations include involvement of PHC clinic managers in decision-making, adequate support from senior management and improvement of internal communication practices.

**Conclusion:**

The researcher concludes that the senior management team in the district under study should strive to embrace the described recommendations as a strategy to facilitate managers’ compliance to quality standards at PHC clinics.

## Introduction

The National Department of Health (NDOH) of the Republic of South Africa (RSA) (2007:i) states that compliance to quality standards and commitment to maintain these standards should never be incidental but rather the result of a high intention, earnest effort, intelligent direction and skilful implementation by managers at primary health care (PHC) clinics. This statement is deemed strategic and critical for the South African health system to achieve the goal of quality health care for all of its citizens (NDOH 2007). The South African NDOH Negotiated Service Delivery Agreement (NSDA) (2012:4), together with the Gauteng Department of Health (GDOH) (2013:2–3) and the South African NDOH’s Quality Improvement Guide (2013a:1), presented a legislative imperative for compliance with quality standards and view it as a legitimate expectation in a new democratic society, like RSA, particularly at PHC clinics.

The pattern of non-compliance with quality standards at PHC clinics within a district in Gauteng had been evident in the data gathered from audits, such as monthly supervisory visits, quarterly programme in-depth reviews, clinical programme support and the outcomes of national core standards (NCS) audits. In response to this non-compliance, reference materials including policies, strategies and guidelines on clinical programmes were distributed. Together with these, reference materials, management development training, quality improvement workshops, meetings, continuous guidance, support and mentoring through regular support visits were conducted to facilitate managers’ compliance with quality standards. The reports from these support visits indicated that remediation measures, to facilitate managers’ compliance quality standards, were discussed.

Despite the aforementioned implemented interventions, managers’ non-compliance to quality standards at PHC clinics within a district in Gauteng continued to be a reality. Persistence of non-compliance to quality standards is significantly detrimental to the RSA in realising quality health care for all through the implementation of the National Health Insurance (NHI). This predicament necessitated an exploration and description of the reasons for managers’ non-compliance with quality standards at PHC clinics within a district in Gauteng so that recommendations could be described in order. This article focusses only on one part of a larger study, that is, recommendations to facilitate compliance with these quality standards.

## Definition of key concepts

‘Compliance’ is the practice of obeying rules or requests made by people in authority (Oxford Advanced Learner’s Dictionary 2010:116). Compliance will only be facilitated if the criteria of all quality measures are met (SA NDOH 2011b:15). Whittaker et al. (2011:60) define ‘quality standards’ as standards that are used to assess and establish the extent to which an institution meets its clients’ needs and expectations.

## Background

Compliance with quality standards, particularly the NCS, has been identified in the South African NDOH’s Policy Paper on NHI (2011a:31–32) as one of four significant interventions that need to take place for the country to successfully implement the NHI and consequently reduce child and maternal mortality ratios (RSA 2014). With the intention of facilitating compliance with quality standards in health care, the Director General for the South African NDOH (2011b:6) urged for PHC clinics managers who do not comply with quality standards, to undergo a process of continuing enhancement as a strategy to improve outcomes for patients in the country. Furthermore, the Director General for the South African NDOH stated that managers who do not comply with quality standards would be expected to make rapid enhancements in service delivery to attain compliance, or face gradual punitive measures (NDOH 2011b:6). This statement highlights the importance of compliance with quality standards in healthcare establishments, including PHC clinics, as another strategic imperative.

As a crucial step towards a strengthened, effective health system that aims to improve health outcomes and ensure a better life for all South Africans (NDOH 2013a:1), compliance to quality standards by health establishment managers is non-negotiable. Franco et al. (2009:iii–iv) and Oosthuizen and van Deventer (2010:1) declare that a distinctive characteristic of a strong health system is compliance to quality standards with the aim of preventing costly legal actions, complications of sub-optimal care with morbidity and mortality repercussions, improved efficiency of healthcare service provision and patient satisfaction. The South African NDOH’s Primary Health Care Supervision Manual (2009:4–5), Flodgren et al. (2011:6) and Nicklin (2014:1) affirm that compliance with quality standards will ensure a decrease in the healthcare errors, improve patient outcomes and efficiency and effectiveness of healthcare facilities.

The South African NDOH (2016:14) reported on the baseline NCS standards audit, which was conducted in 2011–2012, followed by the ensuing inspections from the Office of Health Standards Compliance (OHSC). These baseline audits and inspections revealed undesirable results for the health establishments, because this non-compliance with quality standards could be viewed as disregarding human dignity, poor client satisfaction and the quality of clinical diagnosis and treatment (GDOH 2013:2–3). The Ideal Clinic programme is another initiative that ensures systematic quality improvement and corrects identified deficiencies in PHC clinics (SA NDOH 2016:14). This Ideal Clinic strategy addresses various components that are required to ensure that PHC facilities in the RSA comply with the quality standards (NDOH 2016:14). These components as described by the South African NDOH (2016:14) include good infrastructure, adequate staff, adequate medicine and supplies, good administrative processes and adequate bulk supplies that use applicable clinical policies, protocols, guidelines and partner and stakeholder support.

## Research design and methods

This study utilised a qualitative, exploratory, descriptive and contextual research design. Twelve in-depth, semi-structured interviews with managers of PHC clinics were conducted within a district in Gauteng.

### Population and sampling

The population of the study comprised managers of the 82 PHC clinics within a district in Gauteng. A purposive sampling method was used in this study. Those who had 5 or more years of experience in managing a PHC clinic, who had undergone a quality standards audit and who were willing to participate in the study were included in this study. The sample size of 12 managers who participated was determined by data saturation.

### Data collection

An experienced interviewer well-versed with qualitative research conducted the in-depth, semi-structured individual interviews. As a quality standards compliance officer in the district under study, the researcher did not participate in the data collection as this might result in bias and the Hawthorne effect. The date and time were fixed (allowing 30–45 min per individual interview). However, the interviewer had to be flexible to ensure that participants were given sufficient time to share their perspectives on how best their compliance to quality standards could be facilitated. The venue for the interview was prepared and communicated to the participants in advance and was agreed upon by all parties. The interviewer held a briefing session with the participants before the actual interviews to facilitate rapport.

The question posed to the managers in an attempt to address non-compliance to quality standards at PHC clinics within a district in Gauteng was:

What can be done to facilitate managers’ compliance with quality standards in this clinic?

After obtaining permission from the participants, the interviews were recorded using an audio tape recorder, and the information was transcribed verbatim.

### Data analysis

The data obtained during the in-depth, semi-structured individual interviews from the PHC clinic managers were analysed using Tesch’s method of analysis for qualitative data (Tesch 1992:117 cited by Creswell, 2013:198). These steps entailed organising and preparing data for analysis, reading and rereading through data to obtain a general sense of the information and its overall meaning and then coding data according to themes and sub-themes. The researcher and the independent coder held a consensus discussion meeting to agree upon the themes and sub-themes that emerged from the data analysis.

### Ethical considerations

The Faculty of Health Sciences Academic Ethics Committee (REC-01-151-2015) at the University of Johannesburg and Research Ethics Committee (15/05/2015-3) of the district under study granted ethical approval for the study. The following ethical principles were adhered to throughout the study: respect for persons; the principle of justice; and the principle of beneficence (Dhai & McQuoid-Mason 2011:14–15). Voluntary written informed consent was requested from the participants after a thorough explanation on the purpose and the method of the study. Participants were also requested permission to use a tape recorder during the interviews. To ensure privacy and confidentiality, participants were coded. The researcher, study supervisor and independent coder are the only people who had access to the audiotapes and transcriptions thereof. Although time for data collection was fixed (allowing 30–45 min per individual interview), the interviewer had to be flexible to ensure that participants were given equal time to share their reasons for non-compliance with quality standards. There were no risks envisaged from participating in this study, rather recommendations were to be described to facilitate managers’ compliance with quality standards at PHC clinics within a district in Gauteng.

## Results

One central theme with two main themes emerged from the data analysis. However, for the purpose of this article, only recommendations pertaining to sub-themes of Theme 1 (challenges with management practices) will be described. These include:

- Involvement of PHC clinic managers in decision-making by senior management
- Implementation of effective and functional support systems to facilitate compliance with quality standards
- Improvement of internal communication practices to facilitate compliance with quality standards.

## Discussion

Based on the challenges with management practices described above, the following recommendations were described. These recommendations, aimed at the local area managers, sub-district and district managers within a district in Gauteng were informed by the participants’ propositions and were integrated into relevant and international literature to add credibility and meaning.

### Involvement of primary health care clinic managers in decision-making that facilitates compliance with quality standards

Participants indicated that they should be involved and consulted whenever important decisions pertaining to their clinics are made. This is evident in the following statements:

‘We should be involved in decision-making and not only be invited to meetings which are about what the principals want and what they have decided already.’ (P9, P10)

‘I am not saying management should always accept what we have to say, but we must be involved in decision-making.’ (P10)

‘As a clinic manager, I need to be involved when decisions are being taken, for example, when interviews are being conducted for lower categories, I must be invited.’ (P4)

Quagraine (2010:48, 68, 69) points out that employee involvement in decision-making is a good practice, with benefits to be gained from it, such as a highly positive and favourable impact on the implementation of policies. The author further asserts that employees’ views should be sought on matters that affect their work, and that they should be empowered to take decisions, which will result in them developing a strong sense of self-worth and a feeling of belonging. Involvement in decision-making creates an enabling environment for creativity and growth because employees who see themselves as stakeholders are motivated to give their best in their organisation and to strive for compliance with quality standards.

Nooritajer and Mahfozpour (2008:604) state that organisational theorists in the field of management believe that senior managers should allow lower level managers and other employees to participate in decision-making, not just for creative decisions, which are accepted and executed more easily by those allowed to participate, but also to increase productivity.

The researcher believes that senior management within the district under study can facilitate managers’ compliance with quality standards by engaging PHC clinic managers in decisions taken, particularly those decisions regarding issues that affect the clinics’ day-to-day operations. Bhuiyan (2010:123) and Irawanto (2015:159) support this notion and stipulate that managers should be involved in collaborative decision-making to influence the organisational effectiveness for compliance.

### Implementation of effective and functional support systems to facilitate compliance with quality standards

Having articulated a lack of support from the senior management within the district under study as a reason for non-compliance with quality standards, PHC clinic managers announced a need for further support from senior management, including guidance and assistance to comply with quality standards.

This is evident in the following statements:

‘If one could receive support from the principals (top management), that would make a lot of difference.’ (P3)

‘I do not have a diploma or a degree in management, but I am a clinic manager and I need to be supported.’ (P11)

The Canadian Nurses Association (CNA) (2010:3) stipulates that having the support of a nurse manager–which in the context of this study can be an area manager, nursing service manager, nursing sub-district manager or the nursing district manager–is of paramount importance to enable personnel in healthcare delivery to function better. This support is related to a more positive ethical working environment, which promotes compliance with quality standards (CNA 2010:3).

The senior managers in healthcare institutions have to provide support to employees with the purpose of enabling them to achieve set objectives, such as compliance with quality standards (Brown et al. 2013:465; Effken et al. 2010:190; Munyewende, Rispel & Chirwa 2014:7)

For the PHC clinic managers to comply with quality standards, they require functional support systems that enable people management and work environments, as this is fundamental for high quality care and good health outcomes (Dixon-Woods et al. 2014:106; SA NDOH 2013b:38).

The implementation of policies and legislations, such as the NHI, requires all health establishments to comply with quality standards, including the NCS. Helfrich et al. (2007:279) believe that effective implementation of such policies can only be facilitated by management’s commitment and support. The researcher believes that this will create a favourable implementation climate for compliance with quality standards at PHC clinics within a district in Gauteng.

While participants cited the need for general support from senior management to assist participants to comply with quality standards, some participants were specific about the type of support they required, namely, (1) support with standard operating procedures and quality improvement plans and (2) support at clinic visits.

#### Support with standard operating procedures and quality improvement plans

This is evident in the following statements:

‘I should be assisted and supported by management on how to draft and compile some documents such as standard operating procedures (SOPs) at an operational level.’ (P11)

‘Management should come to the clinic to assist and support us comply with the quality standards, and ensure that things like quality improvement (QI) plans are compiled.’ (P7)

‘If I inform my manager that [a] hand-washing campaign or patient satisfaction survey is to be held on such and such a day, the manager should come to support and ensure that we doing it the correct way.’ (P7)

McInnes et al. (2014:3) state that the strategy considered important by many participants towards compliance with quality standards–such as patient safety and infection control through proper and regular hand washing–was a top-down approach towards embedding best practice at all levels of the organisation. Furthermore, this can be achieved by making compliance with quality standards part of the organisational mantra, whereby senior managers visibly champion and mandate best practice initiatives, and articulate that non-compliance is culturally and professionally unacceptable (McInnes et al. 2014:3)

Tucker and Singer (2013:30–31) conclude that organisations whose managers ensured that problems were addressed, achieved better results. This suggests that improvement programmes are more likely to change employees’ perceptions when they result in action being taken to resolve problems, as opposed to when they are nothing more than a symbolic show of managerial interest. McInnes et al. (2014:2) and Parand et al. (2014:5) state that senior managers’ support and involvement play a vital role, more specifically in hand hygiene. Input into the design and operationalisation of QI programmes, such as hand-washing audits and campaigns, are beneficial to safety performance and quality standards’ compliance. McInnes et al. (2014:2) further state that senior managers should be present at such activities, not just to support staff but also to offer a perspective on how to improve areas of practice that healthcare organisations often find challenging.

It is evident that participants require management support when compiling the required QI documentation and supporting QI initiatives. In addition, senior managers’ attendance and support during hand-washing audits were suggestions made to facilitate managers’ compliance with quality standards at PHC clinics within a district in Gauteng.

#### Support at clinic visits

Participants stated that senior management needed to show their support by being present at PHC clinics for supervisory visits at least weekly, and by being actively involved in their staff’s needs.

This is confirmed in the following statements:

‘Management must support my clinic, not just by talking, but by doing as well. They need to conduct regular support visits to the facility, ensure that they come down and ensure that we have all what we need.’ (P7)

‘It’s not like we expect management to hold our hands every step of the way, but they should come to the facility for support at least once a week.’ (P4, P7, P9)

‘With constant supervision and support, whereby our managers come to the clinics on a regular basis, we can move towards compliance with quality standards.’ (P5, P7, P9)

Sullivan and Wyatt (2006:47) argue that owing to apparent failures to ensure adequate quality patient care, societies demand that supervision of healthcare services be improved. This is further supported by the International Council of Nursing (ICN), as described in Bauman (2007:2), who asserts that practice environments that offer professional and managerial support can influence individual performance and compliance with quality standards.

Mann (2009:15–26) and Tucker and Singer (2013:2) propose that senior managers need to demonstrate their commitment to quality standards, including patient safety in a visible fashion, for instance, by visiting healthcare clinics in the form of ‘Executive Walk Rounds’ or Management By Walking Around (MBWA). The MBWA can be considered active involvement as opposed to the mere physical presence of senior managers, and enables senior managers to work with staff to identify and resolve problems; this positively influences personnel to comply with quality standards (Mann 2009:15–26; Tucker & Singer 2013:2).

West et al. (2011:n.p.) suggest that when healthcare staff feel that their work environment is positive and supportive, as evidenced by coherent, integrated and supportive people management practices, there are low and declining levels of patient mortality as a result of compliance with the quality standards of health care. Lee and Cummings (2008:773) report that managers in healthcare facilities who experience organisational management support are likely to be committed to quality standard compliance.

Birken, Daniel Lee and Weiner (2012:3) assert that senior managers who were involved and who committed themselves to support QI efforts at their healthcare institutions influenced compliance with quality standards. This is further confirmed in Aarons (2006:1162–1163), who reports that compliance with quality standards through QI initiatives and innovation is positively related to senior management support and commitment.

The Policy on Quality in Health Care for the South African NDOH (2007:22) and Munyewende et al. (2014:11) advocate that for health establishment managers to experience a positive practice environment, senior management must undertake formal supportive supervision on a periodic basis. This will facilitate managers’ compliance with quality standards, by ensuring that PHC clinic managers are being supported in solving problems to improve the quality of health care.

Other participants stated that in order for them to comply with quality standards, management should conduct support visits at PHC clinics to show their appreciation for the good work being done, rather than only visiting clinics when mistakes were made.

This is evident in the following statements:

‘It would be nice to have my manager coming to me just to say, ‘Thank you, you are doing very well’. Manager[*s*] should not only come to the clinic when there are problems, but rather come to show appreciation.’ (P4)

‘I want my manager to be there for me, she must come and support and not only come when I have [*made*] mistakes.’ (P9)

Participants at the Celebrating Innovative Health Management Conference of 2011 (Daire & Gilson 2011:76) articulated that healthcare facility managers are willing to address the challenges in the health system. However, support from the senior management team is paramount at all times, and not only when there is a crisis, such as non-compliance with quality standards wherein fingers are pointed, as in the context of this study (Daire & Gilson 2011:76).

The following statements indicate a need for management support and how this assistance will influence health workers other than the PHC clinic managers to comply with quality standards.

Participants said:

‘Management needs to be more supportive by ensuring that compliance with the quality standards becomes a shared responsibility and not my responsibility alone.’ (P11)

‘Presence of managers at the clinic will show staff that compliance with the quality standards is a requirement and not the clinic manager’s agenda.’ (P11)

In support of the participants’ statements, McFadden, Henagan and Gowen (2009:390) state that managers’ presence at the frontline sends a visible signal that the organisation is serious about resolving problems. This increases employees’ belief that leadership values improvement, which in turn spurs employees to engage in the discretionary behaviours necessary for process improvement. The healthcare managers do not function as independent entities, but within a greater health system; hence, they need to be supported and guided to reach the organisational goals (Engelbrecht & Crisp 2010:199).

Mosadeghrad (2014:86) suggests that to achieve compliance with healthcare standards, supportive management applying techniques and tools to operationalise the quality management constructs can facilitate quality standards. The author proposes that supportive management can influence the clinic manager’s commitment to comply with quality standards.

Other participants articulated the need for support from senior management to comply with the NCS:

‘Getting enough support from our supervisors would assist, by helping and being with us in this boat of National Core Standards, especially with the things that I struggle with as a clinic manager.’ (P12)

‘We should hold working meetings and go through all the domains in the National Core Standards with our supervisor, and then readily get clarity on things that we do not understand.’ (P12)

Parand et al. (2014:1) states that senior healthcare managers have a legal and moral obligation to ensure a high quality of patient care and should strive to improve care by supporting the healthcare institutions, and these managers are ideally located to mandate procedures and organisational climates conducive to compliance with quality standards.

The Policy on Quality in Health Care for the South African NDOH (2007:8) and the Clinical Audit Criteria and Guidance Working Group (2008:7) stipulates that healthcare managers can help to improve compliance with quality standards through supportive behaviour on QI, by identifying weaknesses in the PHC system and by making adjustments for compliance, where necessary.

Participants stated that senior management within the district under study should ensure that adequate support is given to PHC clinic managers by being visible at the clinics and helping them to comply with quality standards. Therefore, it is concluded that the senior management is duty bound to support the PHC clinic managers to enable them to comply with quality standards.

### Improvement of internal communication practices to facilitate compliance with quality standards

Internal communication is a subset of effective organisational communication, which is built upon this simple foundation: communication is a dialogue, not a monologue – it is therefore the dialogic process or conversation between employees and employer and employees and employees (Hopkins 2006:n.p.). Yeomans (2009:334) describes internal communication as an organisation’s managed communication system where employees are regarded as a stakeholder group and are communicated with through a variety of methods, including newsletters, meetings, intranets and feedback sessions.

The managers recommended that poor internal communication practices such as lack of time, or having limited time, to conduct feedback sessions and staff meetings at PHC clinics within a district in Gauteng need to be improved in order to comply with quality standards. They stated that the time to hold feedback sessions and meetings with staff to share new information and discuss staff issues should be allocated.

This is confirmed in the following statements:

‘We must be allocated time for meetings to give feedback to our staff.’ (P11)

‘We can give feedback to our staff if we [are] allowed to meet on a monthly basis.’ (P4)

In support of what the participants said, Booyens (2008a:275) proposes that effective communication can be ensured via feedback sessions where suggestions can be made and opinions and information can be exchanged on how to improve the situation. Furthermore, feedback on general work performance, that is, outcomes of the NCS audit, helps the employees to aim for improvement, set measurable targets with specific deadlines and employ methods to attain them (Booyens 2008a:275). Sufficient managerial feedback to employees is of utmost importance to improve productivity and performance and ultimately to provide quality patient care (McGilton et al. 2006:35; Smith et al. 2007:366–367).

Other participants said:

‘There should be time when I can say just for an hour on a Thursday’ [*I’m*] not going to see patients, but [*I*] have a meeting’. Although this proposal has been declined by management before, it will really help us a lot, because then I can introduce my staff to new things.’ (P10)

‘It is very essential that I hold meetings with my staff and highlight issues and deal with issues from the staff as well, because if I don’t hold meetings then it’s a problem.’ (P12)

‘I should be allowed to have meetings with staff, otherwise the staff dissatisfactions and problems may go unnoticed by me as a clinic manager.’ (P12)

Brown et al. (2009:1219) stipulate that having both regular and scheduled team meetings provide teams with a forum in which to discuss relevant issues, that is, non-compliance with quality standards, in the context of this study, and to solve problems regarding clinical and administrative issues. Furthermore, team meetings are fundamental to the formal communication, and they provide an opportunity to engage all members in consensus-building process (Brown et al. 2009:1219).

The importance of regular team meetings and enhanced communication is highlighted by Xyrichis and Lowton (2008:148–149) as an encouraging work environment for effective teamwork, resolving inter-professional conflict, promoting positive interpersonal relations and encouraging greater levels of innovation, which, according to the researcher in this study, are important for compliance with quality standards. Finch, Hansen and Alexander (2010:18) state that there is no substitute for personal, face-to-face exchange and that managers should be enabled to hold regular meetings with their workgroup.

Regular scheduled meetings for all employees to contribute to practice discussions allow teams to determine positive changes in a practice and to sustain those changes over time. These are described as ‘safe places where employees can raise the issues and receive respectful, collaborative, problem-solving responses’ (Roth & Markova 2012:147). Davies, Homfray and Venables (2013:5) report that effective communication between facility-level staff often ameliorates frustration arising from inadequate communication between senior management and ground-level staff. Regular meetings provide a vital opportunity to encourage others and to resolve programmatic issues, and often act as regular case-based training meetings that increase the nurses’ knowledge and confidence, and allow the nurses an opportunity to debrief with their managers (Davies et al. 2013:5).

The participants in this study articulated that they lacked time to communicate both necessary and relevant information through feedback sessions. Therefore, the researcher can infer that because of these poor internal communication practices, interaction among health workers at PHC clinics within a district in Gauteng is poor; and as such, non-compliance with quality standards, that is, patient safety and poor health outcomes, is likely to result. Adequate time for staff meetings and feedback sessions must be granted to enable health workers to collaboratively identify and address work problems together.

## Limitations of the study

Although this study provided significant findings, the study used a qualitative research approach using only managers and no other healthcare workers in a PHC clinic. Therefore, only qualitative data that are not multifaceted and that represent only the managers’ views regarding non-compliance with quality standards could be obtained from the participants. The results of this study are not transferable as the study was contextual in nature. The district under study has 82 PHC clinics, and the GDOH manages only two of them, while the local government authority oversees the rest. The managers of the GDOH clinics could not avail themselves for data collection, and these clinics are subjected to different management dynamics; therefore, the findings of this study cannot be generalised to the entire district.

## Recommendations for nursing practice, nursing education and future research

The following recommendations are based on the findings of the study. These can be applied in areas of nursing practice, nursing education and future research.

- **Recommendations for nursing practice:** Review and strengthen the existing nursing policies to lead, influence and direct compliance with quality standards at PHC clinics. Incorporate quality-related aspects as a fixed agenda point for nursing management meetings in order to identify practical and realistic measures that can be implemented to enable compliance with quality standards.
- **Recommendations for nursing education:** Integrate the described recommendations meant to facilitate compliance with quality standards in the curriculum for the training of nurse managers in order to have skilled prospective senior nurse managers to ensure effective implementation of management practices such as participative decision-making, employment of effective staff supportive systems and promotion of internal communication practices.
- **Recommendations for future nursing research:** Elicit the perspectives of other health workers, that is, cleaners, enrolled nursing assistants and others, on how compliance with quality standards at PHC clinics, which is a team effort, can best be facilitated. Replicate similar study in another context to obtain different perspectives regarding facilitation of managers’ compliance with quality standards at PHC clinics.

## Conclusion

The proposed recommendations to facilitate compliance with quality standards at PHC clinics within a district in Gauteng were presented as the findings in this study. These results have specific practical implications for the senior management team within the district under study, whereby this team is challenged to implement effective management practices informed by the proposed recommendations as a strategy to facilitate compliance with quality standards.
